# Supplementary material for: Sequentially bidirectional gastrovascular flows in intricately branched digestive tract of planocerid flatworms
Source: PLoS One. 2024 Dec 19;19(12):e0315838. doi: 10.1371/journal.pone.0315838 (PMC11658467; doi:10.1371/journal.pone.0315838)
Supplement: S1 Table — The acquired time of the events from the start (0.3 second) to the end (85.1 second). (DOC) [file pone.0315838.s003.doc]

|  | The percentage of stained tract area in selected ROI (%) | | | |
| --- | --- | --- | --- | --- |
| The timing of the events | **n** | **n+1** | **n+2** | **n+3** |
| 0.3 | 49.28 | 80.17 | 72.82 | 59.69 |
| 1 | 57.28 | 78.97 | 71.89 | 59.93 |
| 1.7 | 63.36 | 79.48 | 70.59 | 59.11 |
| 2.3 | 66.27 | 80.41 | 74.57 | 60.1 |
| 3 | 71.41 | 86.59 | 74.53 | 61.15 |
| 3.7 | 75.56 | 84.66 | 77.25 | 51.33 |
| 4.3 | 75.36 | 89.19 | 76.98 | 50.97 |
| 5 | 76.44 | 85.79 | 76.93 | 49.25 |
| 5.7 | 76.64 | 84.38 | 68.63 | 42.8 |
| 6.3 | 80.43 | 85.75 | 61.15 | 44.57 |
| 7 | 79.36 | 82.56 | 53.76 | 39.28 |
| 7.7 | 80.38 | 84.73 | 52.08 | 31.54 |
| 8.3 | 82.82 | 82.81 | 44.49 | 26.69 |
| 9 | 82.84 | 81.67 | 41.28 | 24.56 |
| 9.7 | 79.1 | 83.33 | 41 | 26.05 |
| 10.3 | 55.53 | 75.83 | 62 | 36.74 |
| 11 | 38.73 | 64.43 | 70.29 | 48.46 |
| 11.7 | 39.84 | 62.32 | 66.98 | 46.55 |
| 12.3 | 37.77 | 67.14 | 65.6 | 44.09 |
| 13 | 51.47 | 68.21 | 64.86 | 45.15 |
| 13.7 | 54.28 | 72.15 | 70.34 | 47 |
| 14.3 | 56.14 | 73.15 | 71.73 | 47.62 |
| 15 | 60.32 | 77.1 | 75.23 | 50.59 |
| 15.7 | 61.01 | 78.79 | 72.32 | 51.71 |
| 16.3 | 61.16 | 76.88 | 70.6 | 52.63 |
| 17 | 62.84 | 77.91 | 76.7 | 52.23 |
| 17.7 | 64.88 | 80.47 | 74.92 | 51.31 |
| 18.4 | 64.83 | 80.47 | 77.01 | 52.38 |
| 19 | 67.82 | 79.91 | 79.09 | 51.89 |
| 19.7 | 70.24 | 81.55 | 77.28 | 51.48 |
| 20.4 | 72.55 | 82.21 | 71.88 | 46.55 |
| 21 | 73.81 | 81.1 | 70.08 | 46.39 |
| 21.7 | 75 | 83.27 | 68.01 | 41.83 |
| 22.4 | 75.61 | 81.95 | 61.98 | 41.18 |
| 23 | 76.41 | 82.15 | 53.98 | 35.66 |
| 23.7 | 76.03 | 83.16 | 53.78 | 34.03 |
| 24.4 | 74.61 | 81.23 | 41.69 | 29.64 |
| 25 | 78.88 | 83 | 43.33 | 30.57 |
| 25.7 | 80.54 | 83.53 | 40.7 | 27.79 |
| 26.4 | 82.96 | 82.41 | 33.2 | 19.23 |
| 27 | 66.92 | 78.1 | 44.84 | 26.18 |
| 27.7 | 38.68 | 74.84 | 67.59 | 42.52 |
| 28.4 | 30.9 | 62.12 | 62.39 | 47.97 |
| 29 | 32.99 | 61.53 | 57.07 | 47.73 |
| 29.7 | 37.15 | 66.06 | 59.24 | 48.74 |
| 30.4 | 38.69 | 69.36 | 60.32 | 50.08 |
| 31 | 44.37 | 73.93 | 62.74 | 49.8 |
| 31.7 | 46.28 | 75.17 | 66.18 | 52.23 |
| 32.4 | 46.44 | 77.87 | 69.25 | 52.29 |
| 33 | 53.85 | 77.94 | 70.99 | 53.72 |
| 33.7 | 52.62 | 79.14 | 73.38 | 54.74 |
| 34.4 | 52.95 | 78.84 | 71.99 | 54.56 |
| 35 | 57.97 | 79.89 | 72.99 | 53.66 |
| 35.7 | 58.03 | 81.25 | 77.39 | 55.4 |
| 36.4 | 60.93 | 80.95 | 77.31 | 52.7 |
| 37 | 60.48 | 81.4 | 79.24 | 55.34 |
| 37.7 | 62.34 | 83.12 | 80.09 | 55.88 |
| 38.4 | 64.1 | 81.82 | 80.25 | 52.58 |
| 39 | 64.82 | 83.03 | 78.24 | 50.84 |
| 39.7 | 67.72 | 82.46 | 79.63 | 50.96 |
| 40.4 | 69.95 | 81.82 | 76.39 | 49.34 |
| 41 | 69.95 | 84.59 | 72.76 | 47.06 |
| 41.7 | 71.87 | 83.03 | 65.3 | 42.62 |
| 42.4 | 73.07 | 83.52 | 65.45 | 36.79 |
| 43 | 76.14 | 86.93 | 62.05 | 39.8 |
| 43.7 | 81.99 | 84.52 | 52.22 | 36.13 |
| 44.4 | 75.5 | 82.67 | 72.09 | 47 |
| 45 | 63.54 | 74.36 | 70.98 | 46.91 |
| 45.7 | 48.91 | 74.5 | 65.51 | 50.46 |
| 46.4 | 35.86 | 72.3 | 66.91 | 50.3 |
| 47 | 36.76 | 75.39 | 63.04 | 49.82 |
| 47.7 | 35.66 | 74.96 | 58.73 | 47.54 |
| 48.4 | 41.91 | 76.96 | 66.76 | 51.38 |
| 49 | 46.63 | 77.73 | 69.83 | 51.43 |
| 49.7 | 48.96 | 77.88 | 71.44 | 52.46 |
| 50.4 | 45.19 | 80.72 | 71.51 | 53.66 |
| 51.1 | 47.37 | 78.57 | 71.66 | 53.66 |
| 51.7 | 45.51 | 79.8 | 72.68 | 53.54 |
| 52.4 | 45.61 | 79.49 | 74.65 | 55.56 |
| 53.1 | 48.5 | 81.18 | 74 | 52.34 |
| 53.7 | 52.16 | 81.85 | 74.87 | 50.9 |
| 54.4 | 53.96 | 82.6 | 75.16 | 51.71 |
| 55.1 | 51.83 | 83.27 | 74.85 | 49.26 |
| 55.7 | 49.31 | 80.05 | 76 | 46.44 |
| 56.4 | 54.41 | 80.95 | 77.47 | 49.47 |
| 57.1 | 55.36 | 81.75 | 74.54 | 48.46 |
| 57.7 | 55.8 | 79.6 | 76.62 | 48.29 |
| 58.4 | 54.56 | 80.47 | 78.78 | 46.61 |
| 59.1 | 57.64 | 79.25 | 75.46 | 47.39 |
| 59.7 | 60.32 | 80.17 | 77.01 | 46.16 |
| 60.4 | 64.48 | 81.29 | 73.69 | 43.59 |
| 61.1 | 68.06 | 81.36 | 67.21 | 36.97 |
| 61.7 | 72.42 | 77.93 | 64.89 | 46.83 |
| 62.4 | 76.04 | 76.22 | 68.9 | 46.11 |
| 63.1 | 60.52 | 58.63 | 61.03 | 45.21 |
| 63.7 | 30.31 | 47.68 | 60.26 | 46.06 |
| 64.4 | 26.78 | 46.2 | 62.04 | 47.09 |
| 65.1 | 26.93 | 52.79 | 63.5 | 48.06 |
| 65.7 | 25.95 | 54.21 | 63.12 | 48.4 |
| 66.4 | 25.32 | 55.89 | 63.58 | 47.94 |
| 67.1 | 32.12 | 55.56 | 63.04 | 46.29 |
| 67.7 | 30.93 | 60.07 | 64.43 | 50.06 |
| 68.4 | 33.26 | 61.75 | 63.12 | 48.74 |
| 69.1 | 58.07 | 76.09 | 71.37 | 53.54 |
| 69.7 | 68.4 | 79.46 | 73.07 | 51.5 |
| 70.4 | 71.67 | 81.82 | 67.75 | 47.42 |
| 71.1 | 72.18 | 82.74 | 64.58 | 44.06 |
| 71.7 | 70.63 | 82.54 | 68.9 | 45.8 |
| 72.4 | 72.34 | 79.41 | 62.73 | 39.74 |
| 73.1 | 71.56 | 81.13 | 55.17 | 40.74 |
| 73.7 | 73.43 | 81.49 | 49.54 | 32.8 |
| 74.4 | 74.16 | 82.38 | 47.84 | 30.8 |
| 75.1 | 73.17 | 81.25 | 50 | 31.26 |
| 75.7 | 74.99 | 80.95 | 46.91 | 32.34 |
| 76.4 | 76.8 | 81.43 | 46.14 | 30.11 |
| 77.1 | 77.11 | 80.65 | 38.81 | 24.99 |
| 77.7 | 77.17 | 80.06 | 44.06 | 34.9 |
| 78.4 | 76.86 | 79.35 | 43.46 | 51.2 |
| 79.1 | 77.48 | 77.86 | 72.68 | 49.14 |
| 79.7 | 76.36 | 70.24 | 61.36 | 48.2 |
| 80.4 | 53.44 | 48.56 | 57.2 | 48.32 |
| 81.1 | 27.67 | 45.72 | 59.39 | 48.98 |
| 81.7 | 27.53 | 46.57 | 56.76 | 48.74 |
| 82.4 | 26.72 | 50.59 | 59.82 | 52.03 |
| 83.1 | 26.82 | 50.07 | 58.95 | 51.71 |
| 83.8 | 25.06 | 51.52 | 59.75 | 52.55 |
| 84.4 | 25.01 | 47.89 | 60.04 | 52.03 |
| 85.1 | 27.62 | 51.45 | 60.56 | 52.42 |
| n, n+1, n+2, n+3 stand for the consecutive order of tract branches | | | | |
