# Supplementary material for: Sequentially bidirectional gastrovascular flows in intricately branched digestive tract of planocerid flatworms
Source: PLoS One. 2024 Dec 19;19(12):e0315838. doi: 10.1371/journal.pone.0315838 (PMC11658467; doi:10.1371/journal.pone.0315838)
Supplement: S2 Table — (DOCX) [file pone.0315838.s004.docx]

|  | inward flow (seconds) | | | outward flow (seconds) | | |
| --- | --- | --- | --- | --- | --- | --- |
| no. | n | n+1 | n+2 | n+2 | n+1 | n |
| 1 | 17 | 15 | 16 | 15 | 12 | 13 |
| 2 | 19 | 19 | 18 | 10 | 12 | 12 |
| 3 | 15 | 16 | 18 | 13 | 13 | 15 |
| 4 | 20 | 21 | 21 | 13 | 13 | 14 |
| 5 | 21 | 18 | 18 | 12 | 12 | 13 |
| 6 | 16 | 16 | 16 | 14 | 14 | 15 |
| 7 | 22 | 15 | 17 | 15 | 11 | 14 |
| 8 | 17 | 21 | 22 | 11 | 13 | 12 |
| 9 | 18 | 18 | 18 | 12 | 14 | 11 |
| 10 | 21 | 15 | 16 | 13 | 13 | 13 |
| mean | 18.6 | 17.4 | 18.0 | 12.8 | 12.7 | 13.2 |

n, n+1, n+2, n+3 stand for the consecutive order of tract branches
