# Supplementary material for: Sequentially bidirectional gastrovascular flows in intricately branched digestive tract of planocerid flatworms
Source: PLoS One. 2024 Dec 19;19(12):e0315838. doi: 10.1371/journal.pone.0315838 (PMC11658467; doi:10.1371/journal.pone.0315838)
Supplement: S3 Table — (DOCX) [file pone.0315838.s005.docx]

|  | The percentage of stained tract area in selected ROI (%) | | |
| --- | --- | --- | --- |
| The timing of the events | **n** | **n+1** | **n+2** |
| 0.03 | 61.08 | 70.12 | 84.84 |
| 0.1 | 62.84 | 70.68 | 84.88 |
| 0.17 | 60.44 | 68.66 | 85.58 |
| 0.23 | 60.82 | 66.88 | 84.45 |
| 0.3 | 60.54 | 67.58 | 85.55 |
| 0.37 | 61.38 | 69.53 | 85.41 |
| 0.43 | 53.4 | 69.5 | 86.46 |
| 0.5 | 58.49 | 70.24 | 85.78 |
| 0.57 | 56.67 | 68.75 | 85.6 |
| 0.63 | 60.19 | 70.01 | 85.47 |
| 0.7 | 67.95 | 70.5 | 88.16 |
| 0.77 | 74.68 | 68.99 | 83.62 |
| 0.83 | 83.59 | 69.5 | 84.36 |
| 0.9 | 88.17 | 70.84 | 86.42 |
| 0.97 | 85.43 | 71.63 | 86.09 |
| 1.03 | 86.05 | 72.22 | 84.54 |
| 1.1 | 90.57 | 72.68 | 84.86 |
| 1.17 | 89.75 | 71.09 | 86.19 |
| 1.23 | 91.38 | 71.69 | 87.16 |
| 1.3 | 88.88 | 71.9 | 87.59 |
| 1.37 | 79.08 | 72.11 | 87.43 |
| 1.43 | 72.35 | 69.99 | 87.24 |
| 1.5 | 80.26 | 70.67 | 87.57 |
| 1.57 | 72.91 | 71.14 | 87.42 |
| 1.63 | 70.66 | 70.44 | 85.76 |
| 1.7 | 67.6 | 70.83 | 86.68 |
| 1.77 | 67.91 | 70.68 | 86.95 |
| 1.84 | 66.22 | 70.08 | 87.19 |
| 1.9 | 64.44 | 71.28 | 87.27 |
| 1.97 | 64.59 | 72.84 | 87.83 |
| 2.04 | 63.3 | 72.48 | 87.99 |
| 2.1 | 76.02 | 72.83 | 87.83 |
| 2.17 | 74.95 | 73.28 | 87.19 |
| 2.24 | 73.74 | 72.65 | 86.78 |
| 2.3 | 69.54 | 70.67 | 87.04 |
| 2.37 | 69.73 | 70.99 | 87.78 |
| 2.44 | 67.14 | 71.07 | 85.96 |
| 2.5 | 69.2 | 70.59 | 85.31 |
| 2.57 | 68.2 | 70.87 | 86.31 |
| 2.64 | 66.14 | 70.45 | 86.19 |
| 2.7 | 63.64 | 70.87 | 86.35 |
| 2.77 | 66.07 | 70.89 | 86.91 |
| 2.84 | 63.51 | 68.86 | 85.58 |
| 2.9 | 66.48 | 65.97 | 86.93 |
| 2.97 | 63.56 | 68.34 | 87.37 |
| 3.04 | 64.23 | 69.14 | 87.66 |
| 3.1 | 62.22 | 70.47 | 86.81 |
| 3.17 | 71.16 | 70.05 | 88.2 |
| 3.24 | 70.35 | 71.18 | 85.68 |
| 3.3 | 68.01 | 70.32 | 85.37 |
| 3.37 | 63.18 | 71.18 | 85.27 |
| 3.44 | 66.57 | 72.42 | 85.88 |
| 3.5 | 72.21 | 71.59 | 85.14 |
| 3.57 | 75.18 | 71 | 87.14 |
| 3.64 | 69.58 | 70.39 | 85.2 |
| 3.7 | 66.48 | 68.87 | 85.05 |
| 3.77 | 65.28 | 68.87 | 85.63 |
| 3.84 | 67.81 | 67.45 | 85.89 |
| 3.9 | 66.62 | 66.61 | 85.81 |
| 3.97 | 77.43 | 65.07 | 85.81 |
| 4.04 | 73.7 | 63.66 | 85.56 |
| 4.1 | 74.03 | 63.16 | 85.24 |
| 4.17 | 69.97 | 64.12 | 84.92 |
| 4.24 | 72.88 | 65.19 | 84.8 |
| 4.3 | 62.89 | 66.53 | 86.04 |
| 4.37 | 69.25 | 67.08 | 85.11 |
| 4.44 | 67.38 | 67.22 | 86.19 |
| 4.5 | 70.06 | 67.68 | 85.33 |
| 4.57 | 69.87 | 69.72 | 85.75 |
| 4.64 | 73.08 | 68.69 | 87.12 |
| 4.7 | 67.34 | 66.99 | 84.29 |
| 4.77 | 65.76 | 65.3 | 84.67 |
| 4.84 | 65.18 | 64.77 | 85.24 |
| 4.9 | 62.27 | 64.4 | 86.5 |
| 4.97 | 60.5 | 62.32 | 86.92 |
| 5.04 | 63.13 | 60.85 | 86.14 |
| 5.11 | 60.88 | 61.57 | 85.11 |
| 5.17 | 56.77 | 62.43 | 85.83 |
| 5.24 | 51.84 | 64.18 | 86.4 |
| 5.31 | 46.39 | 65.64 | 86.38 |
| 5.37 | 49.12 | 65.53 | 87.1 |
| 5.44 | 54.9 | 67.48 | 88.21 |
| 5.51 | 51.36 | 65.93 | 82.52 |
| 5.57 | 53.42 | 68.71 | 85.25 |
| 5.64 | 63.61 | 69.72 | 85.5 |
| 5.71 | 65.95 | 69.46 | 86.13 |
| 5.77 | 70.83 | 70.95 | 85.11 |
| 5.84 | 79.67 | 71.07 | 85.89 |
| 5.91 | 75.75 | 73.97 | 85.66 |
| 5.97 | 75.47 | 62.06 | 85.09 |
| 6.04 | 72.45 | 58.09 | 86.69 |
| 6.11 | 73.03 | 62.65 | 81.66 |
| 6.17 | 73.41 | 64.52 | 83.73 |
| 6.24 | 73.51 | 66.99 | 84.5 |
| 6.31 | 74.03 | 66.23 | 87.57 |
| 6.37 | 70.44 | 66.23 | 87.32 |
| 6.44 | 81.01 | 67.79 | 88.29 |
| 6.51 | 81.01 | 65.98 | 88.22 |
| 6.57 | 83.5 | 65.9 | 88.05 |
| 6.64 | 81.11 | 67.65 | 86.84 |
| 6.71 | 82.54 | 70.23 | 84.75 |
| 6.77 | 81.97 | 66.2 | 84.75 |
| 6.84 | 81.35 | 69.34 | 83.41 |
| 6.91 | 85.89 | 70.03 | 84.13 |
| 6.97 | 81.68 | 68.92 | 85.12 |
| n, n+1, n+2, n+3 stand for the consecutive order of tract branches | | | |
