# Supplementary material for: Sequentially bidirectional gastrovascular flows in intricately branched digestive tract of planocerid flatworms
Source: PLoS One. 2024 Dec 19;19(12):e0315838. doi: 10.1371/journal.pone.0315838 (PMC11658467; doi:10.1371/journal.pone.0315838)
Supplement: Attachment — Submitted filename: Responses.docx [file pone.0315838.s006.docx]

|  | Line | Reviewer #1: |  |
| --- | --- | --- | --- |
|  |  | It might be worth mentioning in the introduction that simultaneously bidirectional flow suggests ciliary action, while sequentially bidirectional flow indicates muscular action. |  |
|  | reply: | These lines are revised:  They described ciliary motion which was visible in image sequences, and sequentially bidirectional flow or simultaneously bidirectional flow separated by baffles [5]. |  |
| 1 | 92 | What camera was used to capture images? |  |
|  |  | These few lines are added:  For extended video recording, the illuminated transparent glass tank and video camera JVC GZ-RX500BTW, Olympus TG6 were used. Video clips showing the ventral sides of the flatworms were captured after staining to observe the movement of food within the highly branched digestive system. All clips were recorded and transformed into the form of MP4 files as partially shown in S1 Video, and time-lapse video was converted in 2 times play speed as shown in S2 Video. |  |
| 2 | 192 | Feeding apparently stimulates activity of the gastrovascular system. Hydroids behave similarly (see Dudgeon et al., 1999, Biol Bull 196, 1). |  |
|  |  | This section is revised as:  As shown in Fig 8, the activity of the digestive system decreased dramatically after roughly one day and came to a complete stop within two days, after which the remaining food residue was moved back to the pharynx and expelled, in a manner similar to the termination of regular oscillatory behavior of post-feeding in isolated polyps [2] and the defecation reflex observed in hydra [3].  The reference is added in the References section:  **2.** Dudgeon S, Wagner A, Vaišnys JR, Buss LW. Dynamics of gastrovascular circulation in the hydrozoan *Podocoryne carnea*: the one-polyp case. Biol Bull. 1999;196(1): 1-17. |  |
| 3 | 292 | Author contributions section is blank. |  |
|  |  | **Author Contributions**  **Conceptualization:** Po-Chun Hsu and Wei-Ban Jie.  **Data curation:** Po-Chun Hsu and Yu-Hsun Chang.  **Formal analysis:** Po-Chun Hsu, Yu-Hsun Chang and Yu-Ning Chiu.  **Funding acquisition:** No funding.  **Investigation:** Po-Chun Hsu, Yu-Hsun Chang and Yu-Ning Chiu.  **Methodology:** Po-Chun Hsu and Yu-Hsun Chang.  **Project administration:** Wei-Ban Jie.  **Resources:** Wei-Ban Jie.  **Supervision:** Wei-Ban Jie.  **Visualization:** Po-Chun Hsu and Yu-Hsun Chang.  **Writing – original draft:** Po-Chun Hsu, Yu-Hsun Chang and Yu-Ning Chiu.  **Writing – review & editing:** Wei-Ban Jie. |  |

|  |  | Reviewer #2: |  |
| --- | --- | --- | --- |
|  | Line |  |  |
|  |  | It seems that the authors are not aware of some publications in which a similar phenomenon is described in colonial hydroids, whose digestive system is also quite extensive, and the movement of food particles is alternately bidirectional. A list of some publications is given below. |  |
|  | reply: | We are very appreciative of the reminder offered by reviewers. We have read through those references and carefully discussed them. A lot of revisions are based on whose perspective aside just from polyclads'. Thanks. |  |
|  |  | The authors do not provide sufficient evidence to interpret the observed pulsations of the digestive tract segments as peristalsis. It is important to remember that a similar effect of transportation in other invertebrates with a pulsating digestive tract is achieved without peristalsis (see the recommended publications at the end of the review). |  |
|  | reply: | 1. Unlike the segmented movement of hydra, the flatworms exhibited regular centrifugal sphincter contractions inward toward the outermost branches (i.e., highest order end) (Figs 5A, 5B). The organisms then exhibited centripetal movement from the outermost (narrowest) branches back to the pharynx (i.e., first-order digestive tract) (Figs 5C, 5D). 2. This process exhibited a rhythmic pattern. The two supplement information, S1 and S2 Videos are added. |  |
|  |  | The description of food movement through the digestive tract is very superficial: without clarifications and quantitative indicators. The authors of the manuscript do not provide answers to the usual questions:  What is the speed of food movement from the pharynx to the distal parts of the digestive system? How evenly and simultaneously does food move in different branches of the digestive system? How equal in speed are the food supplies to the head and tail ends of the animal's body? How do different branches of the digestive system relate to each other in terms of the speed of food movement? How do different branches of the digestive system relate to each other in terms of the speed of food movement? |  |
|  | reply: | 1. The rare population of polyclad flatworms in the wild is challenging for statistical analysis. 2. We create a new feeding alternative to keep flatworms in the tank longer than ever before. 3. As the result, our preliminary analysis is shown in Fig 7 (S4 Table), the ANOVA of the designated inward flows show no significant differences in consecutive n, n+1, n+2 branches in ANOVA (n=30, d.f.=2, F=0.70, p = 0.50). The designated outward flows also show no significant differences in consecutive n+2, n+1, n branches in ANOVA (n=30, d.f.=2, F=0.39, p = 0.67). |  |
|  |  | The manuscript does not clearly indicate the samples. Or rather, there is no mention of samples at all. |  |
|  | reply: | 1. All the collected specimens in this thesis have been preserved in the Chinese Culture University and geoinformation are also included.  2. The first paragraph of the **Materials and methods** is revised as:  *Paraplanocera oligoglena*, totally 13 samples were gathered from tidal pools in various locations of Taiwan, including 4 specimens from Longdong Bay (25°06'48.1"N 121°55'13.1"E), 6 specimens from Magang (25°01'03.0"N 122°00'00.8"E), and 3 specimens from Houbihu (21°56'19.3"N 120°44'44.1"E). |  |
| 1 | 252–255 | The basis for the assumption about the leading role of the nervous system in regulating peristalsis in many branches (spurs) of the digestive system is not entirely clear.  Thus, in the Discussion section (lines 252–255) it is written: “If diffuse nerve net regulation is indeed required for feeding, then it can be inferred that contractions of the intestinal tract during ingestion may also be regulated by the nervous system. |  |
|  |  | These few lines are revised as below:  If diffuse nerve net regulation is indeed required for feeding, then it can be inferred that contractions of the intestinal tract during ingestion may also be regulated by the nervous system. While the basis for the assumption about the role of the nervous system in regulating peristalsis in many branches of the digestive system is not entirely clear. Meanwhile the integral effect of the magistral hydroplasma flows in colonial hydroid is remaining one of the drive mechanism needed to be considered which might include dynamics of cell movement in the body, pulsations of hydrants and coenosarc, and movement of hydroplasma in the gastrovascular cavity [30].  The reference is added:  **30**. Marfenin NN, Dementyev VS. Integral effect of interaction of parts of a noncentralized biosystem by the example of magistral hydroplasma flow formation in the shoots of colonial hydroid *Dynamena pumila* (L., 1758). Biol Bull Rev. 2024;14(3): 344-359. |  |
| 2 | 255-256 | And further (lines 255-256): "A brain and nervous system with this degree of complexity should have the capacity to coordinate rhythmic contractions in the digestive tract more effectively than hydrozoans".  This statement does not take into account the possibility of coordinating local peristaltic activity without the participation of the nervous system, but only on the basis of hydraulic relationships between different parts of the digestive tract, especially since this has already been described in sufficient detail in hydroids.  I believe that the authors should at least mention an alternative solution to the problem of transporting particles in closed systems. |  |
|  |  | These paragraph is revised as below:  A brain and nervous system with this degree of complexity should have the capacity to coordinate rhythmic contractions in the digestive tract more effectively than hydrozoans. Since the flatworms still exist on all levels of blind sacs distributed between its lowermost and highmost branches, the cause of peristaltic movement of flatworms may also bear the possibility of coordinating hydraulic relationships between different parts of the digestive tract. We infer that the peristaltic movement might function simultaneously with the nerve system and hydraulic relationships between different parts of the digestive tract, especially the hydroplasma flow in colonial hydroids have already been described by Marfenin and Dementyev (2024) in sufficient detail [30]. |  |
| 3 |  | I was unable to find a video in the material proposed for review, although the main conclusions of the future article are based on the video. |  |
|  |  | **Two videos are added in Supporting information**:  **S1 Video. Continuous video recording of *Paraplanocera oligoglena* after feeding with methylene blue dyed baits.**  **S2 Video. Time-lapse video converted from 120 seconds of continuous taping of the *Paraplanocera oligoglena* into 60 seconds clips after feeding with methylene blue dyed baits.** |  |
| 4 |  | Data availability The authors believe that “... all data are fully available without restriction ”, however, the manuscript lacks the primary data from which the authors arrived at the conclusions of the study.  There is no video, no choice with options for staining the subject after feeding, and no choice of digital data from which the graphs are plotted (Figures 6 and 7) |  |
|  |  | **S3 Table** is added to show the data used in the **Fig 6**  **The data set of post-staining video tracking of dyed food passing through consecutive branches n, n+1, n+2, and n+3.** The acquired time of the events from the start (0.3 second) to the end (85.1 second). n, n+1, n+2, n+3 stand for the consecutive order of tract branches.  **S4 Table** for **Fig 7**:  **S4 Table. The data set of contraction periods in consecutive order of the tract branches, n, n+1, and n+2 during the inward and outward flows.**  **S5 Table** for **Fig 8**:  **S5 Table. The data set of variations in tract volume on the 1^st^, 2^nd^, and 3^rd^ days.** |  |
| 5 |  | The reviewer did not find a video from which the authors drew important conclusions. How to check the objectivity of the authors if the material on which they base their conclusions is not presented? |  |
|  |  | **Two videos are added in Supporting information**:  **S1 Video. Continuous video recording of *Paraplanocera oligoglena* after feeding with methylene blue dyed baits.**  **S2 Video. Time-lapse video converted from 120 seconds of continuous taping of the *Paraplanocera oligoglena* into 60 seconds clips after feeding with methylene blue dyed baits.** |  |
| 6 | 96 - 118 | There is no information on samples in the study of food particle transportation and in the study of histological preparations.  On the basis of what number of study objects (specimens of the species) are the results of the study derived? If only one specimen with the most pronounced process of food transport was used for the study, then this should be written about in the manuscript.  In such a case, it is better for the researcher to use an Idiographic Approach (Marfenin, & Dementyev. 2022. Influence of Food Consumption...) If conclusions are drawn from the study of several or many samples, the degree of variation in the process being studied should be clearly described in terms of the of peristalsis, regularity of contractions, amplitude of pulsation, etc.. |  |
|  |  | These few lines are revised as below:  We also captured video clips of the flatworms as stained food passed through the digestive tract. This involved moving the flatworms to the side of a transparent glass tank in front of a backlit white plastic divider providing a uniform light source. Selecting one specimen with the most pronounced process of food transport was used for the study. |  |
| 7 | 116 | What is the error of Matplotlib and Pandas methods ? Has the method been calibrated, i.e., verified its accuracy on images specially created by the authors for known volumes in advance ? Without specifying the error, any method, especially for pattern recognition, is questionable. |  |
|  |  | Percentages of blue in the selected ROI were directly graphed using Matplotlib, creating a preliminary image available in real-time during analysis. Pandas was used to export raw data for subsequent examination and plotting.  These few lines are revised as below:  The lower and upper bounds of the object of interest, in this case, the stretch of tract in the selected ROI, were determined with Otsu’s method [23].  The reference is added:  **23.** Otsu N. A threshold selection method from gray-level histograms. IEEE transactions on systems, man, and cybernetics. 1979;9(1): 62–66. |  |
| 8 | 169 | The authors should clarify the criteria based on which they consider the pulsations they describe to be peristalsis.  How do the authors explain the difference in periods of retraction during pulsations of the digestive tract between inward flows and outward flows? ( see Fig . 7) |  |
|  |  | The explanation of peristalsis has been illustrated in the next second paragraph and **Fig 7** by the authors. For more clarifying it, we revise the paragraph as well as below:  As shown in **Fig 7** (**S4 Table**), the ANOVA of the designated inward flows show no significant differences in consecutive n, n+1, n+2 branches in ANOVA (n=30, d.f.=2, F=0.70, p = 0.50). The designated outward flows also show no significant differences in consecutive n+2, n+1, n branches in ANOVA (n=30, d.f.=2, F=0.39, p = 0.67). The frequency and speed of the contractions remained constant, regardless of the branch level (*n*+1, *n*+2…), which indicates that *P. oligoglena* does not rely exclusively on pharynx contraction for the transport of food. The segmented movements also indicate that the tract itself is involved in the contraction. We observed contractions occurring sequentially from one fixed position to another fixed position, resulting in peristalsis (rhythmic wave-like contractions), first in one direction and then in the opposite direction. Regardless of the direction, the movement remained consistent and sequentially bidirectional, albeit at an uneven rate. This is the first study to describe peristaltic segmented contractions and sequentially bidirectional movement in the gastrovascular cavity of polyclad flatworms.  In **Fig 7**, we also make some revisions:  **Fig 7.** **Contraction period in consecutive order of the tract branches, n, n+1, and n+2 during the inward and outward flows.** The seconds of contraction in order 'n' are marked as circle dots, in 'n+1'are marked as solid squares, and in 'n+2' are marked as triangles. The ANOVA shows that designated inward flows have no significant differences in consecutive branches(n=30, p = 0.50). The outward flows also show no significant differences between consecutive branches (n=30, p = 0.67). |  |
| 9 | 361 | Why does the caption to the picture #1 say specimens and not specimen ? How many samples did you study in total? The manuscript nowhere states the number of samples used in the study.  The manuscript does not end with clear conclusions, i.e. proven statements. For this article, conclusions section would be very relevant. |  |
|  |  | 1. “Specimens” in **Fig 1** is revised as “Specimen”.  2. The first paragraph of the **Materials and methods** is revised as:  *Paraplanocera oligoglena*, totally 13 samples were gathered from tidal pools in various locations of Taiwan, including 4 specimens from Longdong Bay (25°06'48.1"N 121°55'13.1"E), 6 specimens from Magang (25°01'03.0"N 122°00'00.8"E), and 3 specimens from Houbihu (21°56'19.3"N 120°44'44.1"E).  3. The sample used in the study of food particle transportation is replied in question #2 as below:  We also captured video clips of the flatworms as stained food passed through the digestive tract. This involved moving the flatworms to the side of a transparent glass tank in front of a backlit white plastic divider providing a uniform light source. Selecting one specimen with the most pronounced process of food transport was used for the study. |  |
| 10 | 375  Fig 3 | The figure caption does not explain the numerical designations. It is not clear whether these designations refer to a segment of the digestive tract with several sections or only to an individual section of the segment. |  |
|  |  | **Fig 3.** **Consecutive ordering of tract branches from the pharynx toward the distal margin.** The pharynx is indicated as 0, and then the numbers from 1 to 5 correspond to every segment of the digestive tract in each order. |  |
| 11 | Fig 5 | Figure 5 is not sufficient to prove or even illustrate peristalsis. The same effect of pulsations of the digestive tract can occur without peristalsis, as, for example, in colonial hydroids. |  |
|  |  | In **Fig 5**. authors are trying to show the sequential bidirection of flow. The explanation of peristalsis is illustrated in **Fig 7** and we revise it as the reply in the question #8:  **Fig 7.** **Contraction period in consecutive order of the tract branches, n, n+1, and n+2 during the inward and outward flows.** The seconds of contraction in order 'n' are marked as circle dots, in 'n+1'are marked as solid squares, and in 'n+2' are marked as triangles. The ANOVA shows that designated inward flows have no significant differences in consecutive branches(n=30, p = 0.50). The outward flows also show no significant differences between consecutive branches (n=30, p = 0.67). |  |
| 12 | Fig. 6 | Fig. 6 Is it correct to call the “Y” axis “Tract volume” when in fact it is “% of the colored part of the digestive trust” ? |  |
|  |  | The y-axis indicates the percentage of blue area detected within the selected ROI. |  |
| 13 | Fig 7 | Fig. 7 The caption to Figure 7 clearly lacks information: there is no explanation of the symbols: circles, squares, triangles, Could it be variation in sampling? Then it seems to be the only indication of sampling in the manuscript. |  |
|  |  | **Fig 7. Contraction period in consecutive order of the tract branches, n, n+1, and n+2 during the inward and outward flows.** The seconds of contraction in order 'n' are marked as circle dots, in 'n+1'are marked as solid squares, and in 'n+2' are marked as triangles. The ANOVA shows that designated inward flows have no significant differences in consecutive branches(n=30, p = 0.50). The outward flows also show no significant differences between consecutive branches (n=30, p = 0.67). |  |
